# Farmer perception of fall armyworm (*Spodoptera frugiderda* J.E. Smith) and farm‐level management practices in Zambia

**DOI:** 10.1002/ps.5504

**Published:** 2019-07-08

**Authors:** Monica K Kansiime, Idah Mugambi, Ivan Rwomushana, Winnie Nunda, Julien Lamontagne‐Godwin, Harrison Rware, Noah A Phiri, Gilson Chipabika, Mathias Ndlovu, Roger Day

**Affiliations:** ^1^ CAB International, Africa Regional Centre Nairobi Kenya; ^2^ CAB International Egham UK; ^3^ CAB International, Southern Africa Centre (SAC) Lusaka Zambia; ^4^ Zambia Agriculture Research Institute (ZARI) Mount Makulu Chilanga Zambia

**Keywords:** communication, fall armyworm, gender, indigenous practices, invasive, pesticides

## Abstract

**BACKGROUND:**

This paper documents farmer perceptions and management practices for fall armyworm (*Spodoptera frugiderda* J.E. Smith), providing a baseline for the development of sustainable pest management strategies.

**RESULTS:**

91% of farmers correctly identified fall armyworm, and reported it as the most important maize pest during 2016/2017 cropping season, affecting nearly half of cultivated area. Estimated maize yield loss during the season, attributed to fall armyworm was 28%. A majority of farmers (60%) used pesticides for fall armyworm control, along with other cultural/physical practices – hand picking and crushing egg masses/caterpillars (36%), and application of ash/sand in the funnel (19%). Farmers used various pesticide active ingredients, and protective measures were inadequate; >50% of farmers did not use any protective measures while spraying. Significantly more male than female farmers used pesticides (*P* = 0.05), and the reverse was true for cultural practices. Significant maize yield differences (*P* = 0.001) were observed by gender, attributed to differences in utilization of production inputs/practices. At least 77% of farmers received and shared agricultural advice, which can be optimized to spread information on fall armyworm management options.

**CONCLUSION:**

Increased use of pesticides to manage fall armyworm poses health and environmental risks, besides the high cost for farmers and governments. Research into cultural and indigenous practices used by farmers will offer opportunities for alternative and sustainable management practices. Research efforts should pay attention to gender differences in access to resources and inputs. Tackling fall armyworm at the farm level, and averting yield losses will require integrated messaging addressing other production risks. © 2019 The Authors. *Pest Management Science* published by John Wiley & Sons Ltd on behalf of Society of Chemical Industry.

## 1 BACKGROUND

Agriculture provides 60% of all employment in Africa and 80% in rural areas. However, agricultural systems and crop productivity across the continent are under threat of pests and diseases, in particular, invasive species (IS).^1^ Invasives are non‐native species to a specific location that cause multiple impacts to agriculture, environment, and livelihoods.^2^ The accelerated rate of trade and transport has contributed to the problem^3^ and these are likely to drive further biological invasions.^4^ Pratt and Constantine^5^ estimate that just five major invasive species: *Chilo partellus*, Maize Lethal Necrosis Disease, *Parthenium hysterophorus*, *Liriomyza* spp. and *Tuta absoluta*, are causing US$0.9–1.1 in billion economic losses to smallholder farmers across six eastern African countries each year, equating to 1.8%–2.2% of total agricultural GDP for the region. Trade has also been hampered, for example, the presence of an invasive fruit fly (*Bactrocera dorsalis*), has resulted in severe restrictions in the trade of high‐value horticultural produce like mango.

In 2016, a new invasive pest, fall armyworm (*Spodoptera frugiderda* J.E. Smith) (Lepidoptera: Noctuidae), was reported for the first time on the African continent^6^ and spread to 44 countries by 2018.^7^ The pest is native to the Americas and has been reported to feed on up to 186 host plants,^8^ while a thorough literature review and additional surveys reported 353 host plants.^9^ The main hosts have been reported to be maize, wheat, sorghum and rice which constitute the main staple food crops for most countries.^10^ Most of the economic damage is caused by late instar larvae that bore into the maize cob to feed on the kernels.^11^ The potential maize yield reduction due to this pest in Africa has been estimated to range from 8.3 to 20.6 tons per year if management measures are not instituted.^12^ Recent environmental suitability modeling using temperature and precipitation data on fall armyworm life‐history, combined with data on native and African distributions suggest the pest is likely to become resident, infesting maize fields whenever a new crop is grown.^13^ The suitable agro‐ecological conditions in Africa makes fall armyworm a major threat to food security in the region thus requiring effective management methods.^10^

In Zambia, maize is a key staple crop, providing both food and income to the majority of the rural population.^14^ At least 1.5 million smallholder households cultivate maize, which occupies 54% of the 1.4 million hectares of land under crop production. The government spends over 60% of the annual public expenditure on agriculture on maize input and output subsidies.^14^ Over the last 6 years, Zambia has become a surplus maize producer making this crop key for the country in accelerating agricultural‐led economic growth.^15^ The main field pests for maize have traditionally comprised the spotted stem borer, *Chilo partellus* (Swinhoe), maize stalk borer, *Busseola fusca* Fuller and the African pink borer, *Sesamia calamistis* Hampson (Lepidoptera: Noctuidae), of which the most damaging was *C. partellus*.^16^ Consequently, several efforts at biological control conducted in the late 1990s helped to reduce the impact of these pests to low levels^16^ and enabled the country to attain some of her maize yield targets, and be more food secure. With the recent invasion of fall armyworm, Zambia has not been spared. Surveys in seven provinces of Zambia showed the pest is spread across all major agro‐ecological zones, with potential to cause maize economic losses up to US$159 million annually.^7^ At the household level, fall armyworm directly affects capital costs through: increased labor needed to deal with the pest; yield losses; and increased cost of production, all of which affect household incomes.

Crop losses due to insect pests may be prevented, or reduced, by deploying effective crop protection measures, which to a large extent depends on farmers' knowledge and behavior towards pest management, and the availability and effectiveness of crop protection methods.^17^, ^18^, ^19^ It is important to understand what farmers know about insect pests, their perceptions about crop yield damage, the control methods they choose to apply, and the perceived effectiveness of these methods. This justifies the need to survey farmers to obtain insights into the realities which influence pest management decisions, particularly those related to insect pests.

However, most of the available information on what pest management methods farmers are using, and the reasons underlying the use of these methods is currently anecdotal and lacking a solid scientific basis. This study, therefore, was initiated to (i) evaluate farmers' perceptions and practices for management of fall armyworm; (ii) determine maize yield losses due to fall armyworm based on farmer estimates, and factors affecting current maize productivity; and (iii) identify pathways that can be utilized for effective dissemination of information to aid the management of fall armyworm at farm level. Study results provide a critical baseline for programs and the National Agriculture Research and Extension System (NARES) in Zambia by identifying what types of actions might be required for sustainable management of fall armyworm. The information is also crucial in the development of sustainable integrated management strategies for fall armyworm, and other invasive pests.

## 2 MATERIALS AND METHODS

### 2.1 Study area and samples

The study was conducted in Zambia covering three agro‐ecological zones namely: Zone I – Luangwa Zambezi rift Valley; Zone IIa – central, eastern and southern plateau, and Zone III – Northern high rainfall zone. These zones represent diversity in farming systems, agricultural potential, diversity of livelihood strategies (such as fishing, mining), and market access (e.g. cross border trade, urban/peri‐urban). Eight provinces from these zones were purposively selected, targeting those with a known presence of fall armyworm from previous surveys and reports of government extension personnel. The selection of provinces also took cognizance of reported high concentration of maize production.^15^ Sampled provinces were: Northern, Central, Luapula, Southern, Copper belt, North‐Western, Eastern and Lusaka. Nineteen representative districts were identified from which enumeration communities and households were drawn.

The study population comprised maize farmers in the target provinces, from which a representative sample was obtained. The sample size was obtained using Cochran's sample size formula for continuous data.^20^ This gave a sample of 384 respondents from the population, which was adjusted to 450 to mitigate a predicted 20–25% non‐response rate. During the survey, 494 households were interviewed (55% male and 45% female respondents), which added value to the statistical representation of the population. The distribution of the sample size across provinces was done using probability proportional to size method. Systematic random sampling was used to obtain respondent households, targeting every 4th household in the enumeration area. Table 1 shows the sampled agro‐ecological zones, provinces, districts and sample size.

**Table 1:** Study locations and sample size

Agro‐ecological zone	Province	Sample districts	No of respondents	Percentage of female	Percentage of ≤35 years
Zone I – Luangwa Zambezi rift Valley (<800 mm year^−1^)	Lusaka	Chirundu	30	43	21
Zone IIa – central, eastern and southern plateau (800 ‐1000 mm year^−1^)	Central	Serenje, Chibombo, Kapiri Mposhi	77	42	22
	Eastern	Rufunsa, Nyimba, Chipata, Lundazi	100	62	35
	Southern	Mazabuka, Livingstone, Choma	82	40	21
Zone III – Northern high rainfall zone (1000‐1500 mm year^−1^)	Northern	Mbala, Chinsali, Mpika	76	43	24
	Luapula	Mansa	24	38	29
	Copper belt	Mpongwe, Chililabombwe	42	29	7
	North‐Western	Solwezi, Kabompo	63	48	10
	Total		494	45	24

### 2.2 Questionnaire development and delivery

Data were collected during the period from May–June 2018, by administering a semi‐structured questionnaire by trained enumerators after pre‐testing in one sample district. The questionnaire was loaded into the Open Data Kit (ODK) platform, which was used to collect data using tablet computers. Interviews were conducted in the local language of the community during the face‐to‐face interaction. The targeted respondent in a household was either the household head or spouse, or any household member who was responsible for making farming decisions such as crops to cultivate, input use, pest management and sale of produce. Informed consent was sought from the respondents prior to recording their information and the data handled in accordance with the General Data Protection Regulation (GDPR).

The questionnaire focused on: 1) basic information about the interviewee such as gender, age, educational level; 2) maize production practices (varieties grown, yield obtained, external inputs used, key pests and management practices, and chemical use); 3) farmer's knowledge, perceptions and practices with regard to fall armyworm; and 4) farmer sources of information on agriculture and pest management.

Data on maize production was based on 2017, covering production activities in the 2016/2017 cropping season, although yield information was also obtained on the seasons before and after for purposes of understanding any production changes that might be attributable to fall armyworm attack or other factors as perceived by farmers. Maize yield estimate was based on plot‐level data.^21^ This approach is based on farmers estimating harvest from each plot (using own defined units), divided by farmer estimates of the area of the plot (using own defined units), and applied to all plots to obtain yield by the respective households. Data for 2016 and 2017 seasons were based on recall, while for 2018 the yield data was based on actual or estimated yield/prediction where farmers had not harvested yet. This estimation approach has been used in various studies as a direct estimate of average crop yield.^22^, ^23^, ^24^ Local harvest quantity units were converted to kilograms and area units were converted to hectares to ensure comparability across study locations.

The concept of knowledge, perceptions, and practices was used to analyze farmers' pest management decisions. This concept has been widely used in previous studies.^25^, ^26^ In this study, knowledge refers to what farmers know about fall armyworm. Respondents were shown photos of fall armyworm (different stages), including damage /symptoms, and asked if they knew the pest and its local name in their communities. On the other hand, perceptions refers to farmers' perceived pest problem, crop damage, and effectiveness of control measures. Farmers were asked to rate the severity of the fall armyworm based on the proportion of planted area affected. Crop damage quantification was based on farmer estimation of yield before and after fall armyworm attack. Damage quantification by farmers might be less accurate, but it is asserted that this provides good information about farmers perceptions of crop damage,^25^ which in turn conditions their decisions on pest management course of action. Practices refers to actual actions farmers used to control fall armyworm. Farmers listed a number of options, and for those who used pesticides, further information was obtained on pesticides used, dosage applied, spraying regime and how pesticides were handled.

### 2.3 Data analysis

Data were received on an aggregate server in real time, where regular quality checks were done to ensure that the data collected met the required standards. On completion of the field survey, the final datasets were downloaded from the server as CSV files and exported to STATA software for analysis. Descriptive analysis was done by calculating frequencies, means, and standard errors. As much as possible, data were disaggregated by gender and age category for purposes of understanding differences existing among the various categories of farmers. Chi‐square tests were used to compare the significance of categorical variables between farmer categories by age and gender. Analysis of variance (ANOVA) was used for quantitative variables with normal distributions and homogeneous variances. Besides the farmers' perceptions, the factors that may be linked to current maize productivity were assessed using a log‐linear regression model, to understand their effect on maize yield.

## 3 RESULTS AND DISCUSSION

### 3.1 Maize production systems

Maize production in the study area is mostly small‐scale and the average field size was 1.3 ha and claimed a significant proportion of total cultivated land by households (Table 2). Full‐time labor equivalent was 3.4 persons per household. In comparison to the average household size of 7.8 members, this implies that less than half of the household members were engaged in farming activities.

**Table 2:** Maize production characteristics for 2016/2017 cropping season

Characteristic	Male	Female	<35 years	35+ years	Total
Land owned (ha)	5.1	3.0***	3.0	4.5**	4.2
	(0.6)	(0.4)	(0.5)	(0.4)	(0.4)
% cultivated (all crops)	50.8	62.8***	63.0	54.6**	56.2
	(2.5)	(2.2)	(3.1)	(2.0)	(1.7)
Area planted with maize area (ha)	1.5	1.0***	1.1	1.3**	1.3
	(0.5)	(0.1)	(0.2)	(0.3)	(0.3)
Maize area as % of total cultivated area	38.7	51.6***	50.3	42.9**	44.5
	(1.8)	(2.4)	(3.7)	(1.6)	(1.5)
Full time farm labor (Number of members)	3.7	3.2***	2.9	3.6***	3.5
	(0.1)	(0.1)	(0.2)	(0.1)	(0.1)
Agricultural practices used (%)^a^					
Improved seed	91	82***	89	86	87
Inorganic fertilizer	89	83**	88	84	86
Organic manure	45	36**	39	41	41
Maize mono‐cropping	79	73**	71	77**	76
Pesticides	49	40*	41	49	45
Soil and water conservation measures	40	31**	28	38**	36
Average cost of inputs per year/farm (US$):					
Inorganic fertilizers	56	51	44	56*	53
	(2.8)	(6.2)	(3.4)	(3.9)	(3.2)
Pesticides	11.6	4.8**	7.1	8.9	8.5
	(2.3)	(1.4)	(4.2)	(1.4)	(1.4)
Aggregate inputs cost	174	120***	131	154	149
	(13.7)	(11.0)	(18.8)	(10.3)	(9.1)
Total maize harvested (kg)	4738	2548***	3011	3948	3742
	(404.5)	(209.9)	(486.5)	(282.2)	(245.1)
Harvested maize yield (kg ha^−1^)	4753 (262.6)	3097***	3284	4203**	4000
		(204.2)	(305.1)	(205.6)	(174.6)

Figures in parentheses are standard errors.

a Computed based on the number of plots cultivated by farmers. Farmers cultivated multiple plots and often applied different practices based on objective of the crop grown.

Note: ***, ** and * denote significant difference between groups at the 1%, 5%, and 10% significance levels, respectively.

The majority of respondents (87%) planted improved maize varieties. Soil fertility management was mainly through the use of inorganic fertilizers (86% of farmers). Farmers also used organic materials for soil fertility management (41%), mostly incorporation of crop and animal waste to the soil. Farmers cultivated on average three plots of maize per household, primarily in a pure stand (76% of plots). Where intercropping was done, the key crops in the intercrop were; pumpkin, beans, and sweet potatoes. Mixed cropping (more than one crop) was less common and was practiced on just 5% of plots. More than 45% of the farmers used agricultural chemicals ‐ pesticides and herbicides ‐ on their farms. Only 36% of respondents implemented any type of soil and water conservation measures. Main soil and water conservation measures used were soil bunds, minimum tillage, and retention ditches.

Estimated maize production for the 2016/2017 cropping season was on average 3.7 tons, with an average yield of 4.0 tons ha^−1^ (Table 2). Farmers spent approximately US$149 on all production inputs (including labor) during the 2016/2017 cropping season. The expenditure on fertilizers comprised about 36% of all production costs. Expenditure on pesticides was about 6% of total production costs, averaging at $8.5 per year/farm. Production inputs were mainly supplied to farmers through the Farmer Input Support Program (FISP), a government input subsidy program, to which majority of the respondents had participated. Participating farmers in the subsidy program paid a premium to the scheme, averaging $32 to access production inputs (often a package including fertilizer and seed). Besides, farmers mentioned the social security program that also promoted fertilizer use for resource‐constrained farmers.

Production systems varied by age category and gender of the farmer (see Table 2). Male and older farmers owned significantly larger parcels of land compared to female and younger farmers, though the proportion they cultivated was significantly lower. Similarly, the area planted with maize was significantly lower for female and younger farmers compared to their male and older farmers. Female and younger farmers also had significantly less farm labor compared to male and older farmers. Differences also existed for the utilization of agricultural practices. Male farmers were more likely to use improved seed, fertilizer, manure, and soil and water conservation measures compared to their female counterparts. Across the age categories, significant differences were observed in the use of soil and water conservation practices, and cropping system (mono‐crop vs intercrop) only. Aggregate expenditure on production inputs, and specifically expenditure on pesticides was significantly different between male and female farmers, while for inorganic fertilizer expenditure there was no difference, probably linked to the subsidy program to which the majority of farmers participated. Between older and younger farmers, expenditure on inputs was not significant except for inorganic fertilizer. Attained maize yield was significantly lower for female and younger farmers compared to male and older farmers. The greater yields for male farmers could in part be explained by higher use of production inputs, and careful management practices including soil and water conservation.

### 3.2 Fall armyworm perceptions and management practices

#### 3.2.1 Fall armyworm identification and incidence

During the survey, farmers were shown photos of various stages of fall armyworm and symptoms, without the researcher telling them that this was fall armyworm. The majority (91%) of farmers could correctly identify fall armyworm, and 97% (88% of total respondents) had physically observed fall armyworm on their farms during the 2016/2017 cropping season. Farmers also reported it as the most problematic pest during the cropping season, besides other known pests such as aphids, stalk borers and cutworms. Farmers mainly observed the pest on maize, mostly the larval stages (caterpillar), and a smaller proportion also observed the eggs and adult moth. Improved maize varieties were perceived to be more susceptible to fall armyworm compared to local varieties, with the crop being most susceptible at the vegetative stage.

**Figure 1:**
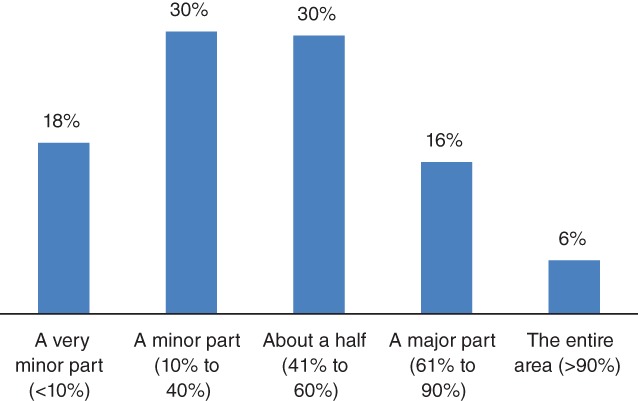
Farmer perception of pest damage in the 2016/2017 season by the proportion of cultivated area.

Asked to indicate the severity of the fall armyworm, for the 2016/2017 cropping season, nearly 50% of farmers reported more than 40% of their cultivated area having been affected with fall armyworm. In terms of pest intensity, the majority of farmers indicated that a minor part (10–40%) or just about half (41–60%) of their maize crop was affected by fall armyworm (Fig. 1). However, 50% of farmers considered fall armyworm less severe in the 2017/2018 compared to the 2016/2017 cropping. Farmers also reported that the pest intensity was generally lower in 2017/2018 cropping season compared to the season before. They attributed this to better pest awareness and early response. Besides, farmers also indicated that they applied fertilizer in time compared to the previous season, which contributed to more effective utilization by the crop. In particular, farmers indicated that in fields where they applied fertilizers, the crop was less affected by fall armyworm compared to fields where they did not use fertilizer. There were no significant differences in farmer perceptions of fall armyworm by gender and age category, as such data disaggregation are not presented.

#### 3.2.2 Fall armyworm management practices

A majority of farmers (62%) used at least one practice for the management of fall armyworm during 2016/2017 cropping season (Table 3). Farmers used various methods for control of fall armyworm that can generally be grouped as; pesticide, cultural/physical, and biological. Pesticide use was the most common method, used by 60% of the farmers. Physical/cultural practices were dominated by hand picking and crushing egg masses (36%), application of ash, sand or liquid detergent on the larvae (19%), and early planting (7%). Use of biologicals was less common, practiced by only 5% of the farmers. Biologicals included farm‐based plant extracts such as neem, tobacco and chili pepper. A few farmers also used biopesticides, in particular, Azadirachtin and Spear (GS‐omega/kappa‐Hxtx‐Hv1a). Use of a combination of methods was common, and a majority of farmers (72%) combined pesticide use and cultural/physical practices.

**Table 3:** Fall armyworm management practices used by farmers

	Gender		Age category		
Fall armyworm management by farmers (%)	Male	Female	≤35 years	35 + years	Combined
Applied any practice^a^					
Yes (n = 321)	62	76**	69	76	73
No (n = 117)	38	24	31	24	27
Practices used (%)^b^					
Pesticides	65	56**	61	59	60
Biologicals	5	5	6	4	5
Physical/cultural practices	30	39**	33	37*	35
Pesticides + cultural practices	73	65**	58	73***	70
Pesticides + biologicals	51	42**	41	49	47
Cultural + biologicals	40	35	30	40**	38
Physical/cultural practices used (%)					
Hand picking caterpillars/egg masses	36	38	41	35	36
Sand/ash	20	19	14	20	19
Early planting	5	11**	0	9**	7
Soil fertility management	4	5	0	5**	4
Destroying infected plants	4	5	2	5	4
Crop rotation	2	5	2	3	3
Intercropping	0	2***	0	1	1

a Computed based on only farmers who observed fall armyworm on their farms in 2016/2017 season (n = 438).

b Computed based on only those farmers who employed any form of control measures (n = 321).

***, ** and * denote statistical difference between farmer categories at the 1%, 5% and 10% levels, respectively.

There were significant differences in the proportion of male and female farmers using various practices for management of fall armyworm. Significantly more male than female farmers used pesticides, while more female than male farmers used physical/cultural practices. More male than female farmers also combined practices, in particular pesticides and cultural or biological products. Across age category, elderly farmers were more likely to employ cultural practices and a combination of cultural and pesticides or biological products than younger farmers.

When asked about the effectiveness of control measures, over 97% of the farmers using pesticides indicated they were effective, particularly if used in alternation. Use of biologicals (farm‐based plant extracts and biopesticides) and early planting were also considered effective despite the small proportion of farmers using them. Physical methods such as hand picking and use of ash were also considered effective by 38% and 54% of farmers, respectively. The only limitation with physical methods as mentioned by farmers was the high labor demand especially for farmers with large plot sizes, rendering them less feasible. However, it was noted that pest management efforts at the local level were often on an individual basis, which may constrain containment efforts. This is due to the migratory nature of the pest, whose population builds up quickly in poorly managed neighboring fields, increasing the farmer's burden towards its management.

#### 3.2.3 Types of pesticides used by farmers

A total of 22 pesticide active ingredients were found to be in use for control of fall armyworm during the survey period. The most common pesticides based on the active ingredients were: Lambda‐cyhalothrin (33%), Cypermethrin (23%), Monocrotophos (9%) and Emamectin benzoate (6%). According to the World Health Organization (WHO), Cypermethrin and Lambda are classified as Class II (Moderately hazardous) pesticides, while Monocrotophos is classified as Class Ib (Highly hazardous). We could not establish the classification for Emamectin, though it is listed as an approved pesticide by the Zambia Environment Management Authority (ZEMA). The pest and disease management guide (PMDG) developed for management of fall armyworm in Zambia specifies only three pesticides – Deltamethrin, Lambda‐cyhalothrin and Emamectin benzoate.^27^ Other pesticides such as Dichlorvos and Methomyl, which are classified as highly hazardous, were also in use albeit by a small proportion of farmers. It is worth noting that farmers also used some biopesticides as well. The most common biopesticides were Azadirachtin and Spear (GS‐omega/kappa‐Hxtx‐Hv1a). Table 4 shows the most commonly used pesticides by proportion of farmers, WHO classification, listing by ZEMA and the most common application rate.

**Table 4:** Pesticides used by farmers for fall armyworm control and their classification

Pesticide name	Active ingredient	Freq.	%	WHO class^a^	ZEMA list^b^	Farmer application rate (gm, mL/16 L water)
Acetochlor	Acetochlor 900 g L^−1^	1	1	III	Y	80
Atrazine	Atrazine 50 SC	1	1	III	Y	4
Glyphosate	Glyphosate 480 g L^−1^ SC	1	1	III	Y	160
Malathion	Malathion 500 g L^−1^	9	5	III	Y	30
Deltamethrin	Deltamethrin 25%	1	1	II	Y	40
Legacy	Chlorpyrifos 48% EC	1	1	II	Y	32
Imidacloprid	Imidacloprid 70% WG	2	1	II	Y	10
Rogor	Dimethoate 400 g kg^−1^	3	2	II	Y	30
Profenofos	Profenofos	4	2	II	Y	16
Cypermethrin	Cypermethrin 20% EC	41	23	II	Y	35
Lambda	Lambda‐cyhalothrin 5% EC	60	33	II	Y	40
Dichlorvos	Dichlorvos	1	1	Ib	N	32
Methomyl	Methomyl	1	1	Ib	N	15
Phoskill	Monocrotophos 400	17	9	Ib	N	30
Abamectin	Abamectin 3.6 EC	1	1	N/A	N	15
Cyclone	Chlorpyriphos 10% + cypermethrin 35%	2	1	N/A	N	10
Spear	GS‐omega/kappa‐Hxtx‐Hv1a	6	3	N/A	N	50
Belt	Fluten Diamide	1	1	N/A	Y	5
Nimbecidine	Azadirachtin	1	1	N/A	Y	50
Alpha 10 EC	Alpha‐cypermethrin 10% EC	3	2	N/A	Y	30
Hitcel	Profenofos 40% + Cypermethrin 4% EC	3	2	N/A	Y	35
Emamectin	Emamectin Benzoate 1.9% EC	10	6	N/A	Y	25

a WHO classification: Ia = Extremely hazardous; Ib = Highly hazardous; II = Moderately hazardous; III = slightly hazardous; U = Unlikely to present acute hazard in normal use; FM = Fumigant, not classified; O = Obsolete as a pesticide, not classified.

b ZEMA list (Y = yes): pesticide recorded on the Zambia Environmental Management Authority list for use in Zambia as pesticide as at 15th June 2018.

Farmers mainly applied pesticides to maize at the vegetative growth stage (88% of farmers), which also corresponds with the stage at which the fall armyworm was mainly observed by farmers. Over 58% of farmers applied pesticides only once during the maize growing season. Those who applied more than once did not have a clear schedule for repeat sprays, as more than 50% sprayed on an ad‐hoc basis. Farmers indicated varied rates of pesticides application based on the product used or stage of the crop. Application rates (mode) for the most common pesticides ‐Lambda, Cypermethrin, and Emamectin – were 40, 35 and 25 g per 16 L of water, respectively. In some instances, farmers mixed chemicals in a single spray or applied higher doses of the same chemical, as they perceived it to be more effective. For example, the maximum dosage for the three most popular pesticides – Lambda, Cypermethrin, and Emamectin – as reported by farmers was 65, 50 and 35 g per 16 L of water, respectively. Farmers also used chemicals in alternation often using a different pesticide (different molecules) if they had to do repeat spraying. Combined with the reported partial application (pesticides being applied on part of a farmer's acreage), it was not easy to establish pesticide use intensity (amount of active ingredient per hectare per year) by farmers.

The use of protective gear was very low, with more than 50% indicating that they did not use any protective wear while spraying chemicals. At least 27% of farmers wore gumboots, while the use of other protective wear such as nose mask, work suit, and gloves were minimal (16%). Asked about health risks, 61% of farmers who sprayed pesticides reported side effects on their health, particularly skin irritation, headaches, and dizziness.

### 3.3 Maize yield losses

**Figure 2:**
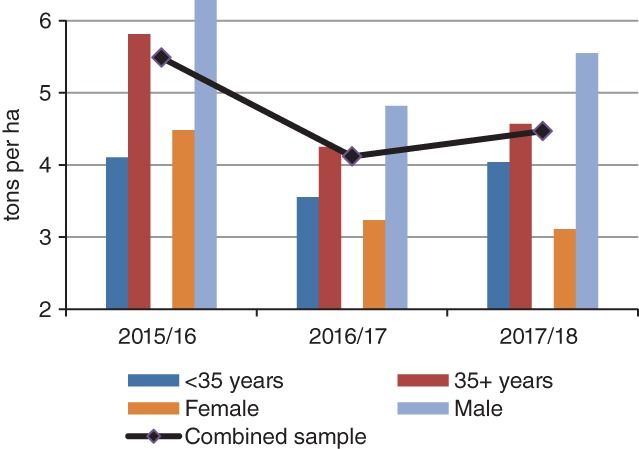
Farmer estimation of the 3‐year maize yield.

Farmers were asked to estimate their maize yield before and after fall armyworm attack in order to estimate the yield loss due to fall armyworm or any other factors that farmers consider key for yield attainment. Comparative analysis of maize production starting from 2015/2016, which was determined to be the season prior to fall armyworm invasion, showed a considerable decline in yield over a 3‐year period, with 2016/2017 season having the lowest yield (4 t ha^−1^) (Fig. 2). The maize yield decline between 2015/2016 and 2016/2017 cropping seasons was estimated at 28%. In the 2017/2018 season, maize yields showed an 11% recovery (overall) from the previous season, although it remained significantly below the average reported in the 2015/2016 cropping season. Farmers attributed the declining yields to fall armyworm attack, drought, and late or no application of fertilizers. In particular, the prolonged drought in 2018 was blamed for the yield decline, despite farmers reporting a marginal decline in fall armyworm incidence during the season.

When extrapolated by gender, there were significant yield differences (*P* < 0.05) between men and women across the three seasons. For the age category, significant differences in yield between younger and older farmers were recorded only for 2016/2017 and 2017/2018 cropping seasons (*P* < 0.1). Female farmers reported a consistent yield decline over the three years. This may imply the effect of other factors on yield other than fall armyworm, such as input use and other farming practices which were significantly different between male and female farmers.

### 3.4 Factors affecting maize productivity

A regression analysis was done to estimate the effects of various socio‐economic and production factors on maize productivity. The dependent variable was maize yield (kg ha^−1^) for the base year (2016/2017) cropping season. Results show that pesticide sprays and the use of cultural practices for fall armyworm management were positively and significantly associated with maize yield (Table 5). This implies that farmers who applied either one or a combination of these methods were likely to have a better yield than those who did not. This may partly explain the farmers' positive perception of the effectiveness of these management methods.

**Table 5:** Factors influencing maize productivity

Log maize yield (kg ha^−1^)	Coef.	Std. Err.
Sex (Male =1)	0.106***	0.037
Age category (35+ years = 1)	0.069	0.045
Labor availability (# full time on farm)	0.031***	0.009
Off farm income (yes = 1)	−0.030	0.076
Access to extension (yes = 1)	0.025	0.068
Access to credit (yes = 1)	0.081	0.052
Farmer group participation (yes = 1)	0.014	0.046
Total livestock units	0.003***	0.001
Farm size (ha)	0.035***	0.007
Pest damage (rank data on a scale of 1–4)	−0.009*	0.016
Pest management – pesticide use (yes = 1)	0.171***	0.052
Pest management ‐ cultural practices (yes = 1)	0.042**	0.023
Pest management ‐ biologicals (yes = 1)	0.038	0.138
Pesticide use + cultural practices	0.219*	0.118
Pesticide use + biologicals	−0.489	0.385
Cultural practices + biologicals	0.546	0.508
Inorganic fertilizer use (yes = 1)	0.277***	0.079
Improved maize variety planted (yes =1)	0.133*	0.087
Constant	2.073***	0.129
No. of observations	484	
F (22, 461)	23.79	
Prob > F	0.000	
R‐squared	0.532	
Adj R‐squared	0.509	
Root MSE	0.388	

Farming practices were not included in the model due to their direct correlation with fall armyworm intensity.

***, ** and * denote statistical difference at the 1%, 5% and 10% levels, respectively.

Farm size and total livestock units showed a significant positive effect on maize yield. These variables relate to a farm's asset endowment which is hypothesized to contribute to better farm management decisions and investments. The pest intensity, as perceived by farmers had a significant negative correlation on maize yield. This implies that the higher the fall armyworm damage, the higher the likelihood of significant maize yield loss. Gender of the farmer and labor availability were positively correlated to maize yield. This implies that male farmers, as well as households with access to surplus labor, were more likely to register higher maize yield. This is also observed from the production trends (cf Fig. 2), where women consistently reported lower yield compared to men.

### 3.5 Farmers' sources of agricultural advice

The study assessed farmers' sources of information and how information is shared, to establish opportunities for communication and engagement with farming communities to manage fall armyworm. All interviewed farmers (100%) reported having received agricultural information during the previous year, from various sources. Farmers information sources were grouped as; local/community groups or external sources. About 29% of farmers relied on own experience and indigenous knowledge (Table 6). Other local sources of information included; neighbors and relatives, farmer field days, cooperative societies, trade fairs, and lead farmers. Government extension officers and programs on radio and TV dominated as the most common external sources of agricultural information. Men were more likely to receive information from extension workers, radio programs and field days compared to women. Women on the other hand, besides extension workers, were more likely to rely on own experience or neighbors/family members for information. Across gender and age category, there were no significant differences in sources of information.

**Table 6:** Farmers sources of agricultural information

	Farmers receiving information from different sources (%)				
Source of information^a^	Women (n = 223	Men (n = 271)	<35 years (100)	35+ years (394)	Combined (n = 494)
*Local and community groups*					
Own experience	33	26	28	30	29
Neighbors and relatives	23	18	22	20	20
Field day	14	20	10	19	17
Cooperative society	18	15	11	18	16
Lead farmer	10	6	3	9	8
Trade fair	2	3	0	3	3
*External sources*					
Extension officer	64	79	64	74	72
Radio/TV	22	34	25	29	28
Plant clinics	4	6	2	6	5
Agro‐dealer	2	7	3	6	5
Magazine	1	3	0	3	2
SMS	4	2	2	3	3

a Multiple sources possible.

At least 77% of the farmers shared the information they received from various sources. The study found that the farmers' mostly shared information with other family members and close‐knit community members. Fewer women than men and fewer younger people than older ones shared information with other farmers. Farmers mainly shared information on the time of planting, crop rotation, pest and disease management, use of inorganic fertilizer and improved varieties.

## 4 DISCUSSION

Effective management of an invasive pest such as fall armyworm is an outcome of two variables: control methods/technologies, and human action. While control methods continue to be refined through scientific research in a systematic manner, there are limited research efforts devoted to the human factor in invasive species management. However, given the diversity in community behaviors and perceptions toward new information and technology, behavioral heterogeneity is a critical issue to understand and predict the success of pest control information diffusion throughout the community.^28^ This study, therefore, documents farmers' perceptions and management of fall armyworm, paving a way for the design of sustainable management strategies for fall armyworm, and consequently invasive species management at a large scale.

Results indicate that a majority of maize farms in Zambia were affected by fall armyworm, in 2016/2017 cropping season having been reported for the first time in the country in 2016. Farmers were able to identify the pest by its physiology or observed damage to the affected plants. A majority of respondents planted improved maize varieties which they also perceived to be more susceptible to fall armyworm compared to local varieties. Thierfelder, Niassy^11^ also report varying susceptibility of maize varieties, with open‐pollinated varieties having lower leaf damage compared to others.

Pesticide use was the most used approach for fall armyworm management, with a prevalent tendency for farmers not to adhere to safety precautions. Since the invasion of fall armyworm, many African governments responded by distributing free pesticides to the affected areas to protect the crops in the field and halt further expansion of the pest. It is therefore not surprising that farmers have continued to use the pesticide approach as the main control method for fall armyworm. In Zambia, the government reportedly distributed more than 100 000 L of pesticide, leading to massive pesticide use in the country in 2016/2017 season, a practice that has continued unabatedly.^29^ Frequent use of synthetic pesticides, however, may have serious implications on the environment, human and animal health. This is because rapid responses such as those that have been deployed for this pest can in most cases lead to the indiscriminate application of pesticides with little regard to safety.^11^ In Zambia, the impacts are likely to be further exacerbated, as we observed for instance that a high proportion of farmers used Monocrotophos, a Class Ib pesticide that is banned in many developed countries. Farmers mentioned that they often used higher than recommended dosages for the most common pesticides, and often combined different pesticides in the belief that the effect will be greater. For example, the fall armyworm technical brief for Zambia recommends use of Lambda cyhalothrin at a rate of 10–15 mL/16 L of water, which is lower than average farmer usage of 40 mL/16 L of water. Technical experts further confirmed this higher than recommended application rates by farmers for most pesticides.^30^ Such high amounts of pesticide use and chemical mixtures have been reported by farmers for management of other pests.^25^ Combined with limited adherence to safety precautions as observed in this study, farmers may be exposed to health risks. Pouokam and Lemnyuy Album,^31^ and Ntow and Gijzen^32^ report significant proportions of acute pesticide poisoning among farmers who generally did not use protective clothing while handling pesticides. It is therefore imperative that farmers are made aware of the risks involved in pesticide handling through the delivery of well‐targeted training programs.^32^, ^33^

Notwithstanding the high pesticide use for fall armyworm management, recent studies in Africa have shown mixed results on the effect of pesticide application on fall armyworm. The findings largely suggest the limited efficacy of pesticides in controlling this pest or reducing its damage and impacts.^11^, ^24^ This has been attributed to wrong pesticides being applied or poor timing of the application. On the other hand, these studies report that cultural practices such as frequent weeding, intercropping, and trap cropping reduced fall armyworm infestation. Midega and Pittchar^34^ report that interactive cropping systems such as push‐pull technology and intercropping of maize with leguminous crops, provide better protection of maize from fall armyworm and other complex pests compared to mono‐cropped maize. In the same study,^34^ it was shown that maize grown in a climate‐adapted push‐pull system had significantly fewer larvae and lower plant damage than maize grown in a monocrop system. According to^10^ intercropping increases plant diversity on the farm, encouraging natural enemies. Use of other non‐chemical practices such as ash/soil and plant extracts have also shown potential with regard to fall armyworm management, besides providing low‐cost options for smallholder farmers.^24^, ^29^ Silva and Broglio^35^ also show that aqueous extracts of neem seed cake were effective for control of fall armyworm on maize in Brazil. These options have low associated health and environmental risks,^11^ and their inclusion in the research efforts for fall armyworm control is important. In particular, considering current farmers' perceptions about their effectiveness on fall armyworm implies the potential for these practices to be promoted to scale for adoption, once validated by research.

The average maize yield loss reported by farmers for the 2016/2017 cropping season, following fall armyworm attack was 28%. In 2017/2018, maize yield showed an 11% yield recovery but was still below 2015/2016 cropping season. Farmers attributed the improvement in yield to better knowledge and early response to fall armyworm compared to the previous season. However, the mid‐season dry spell experienced was reported as a major factor contributing to significant yield drop in 2018 in various maize regions in Zambia (FAO, 2018), despite the reported improved response at farm level and reduced pest severity. Notwithstanding the variations in climatic conditions that directly influence yield, farmers still classified fall armyworm as one of the major drivers of change in maize yield, on a par with drought. This implies a need to effectively tackle the fall armyworm invasion by not only providing sustainable control methods to farmers but taking into consideration other options to manage production risks such as climatic variations.

Across gender and age category, farmer perceptions with regard to fall armyworm control were comparable, though management practices and attained maize yield were significantly different between male and female farmers, and between older and younger farmers. Peterman and Quisumbing^36^ report similar results from Nigeria and Uganda, where female‐owned plots and female‐headed households consistently reported lower productivity, attributed to a range of socioeconomic variables, agricultural inputs use, and crop choice. Croppenstedt and Goldstein^37^ report that these gender differences in land productivity are primarily linked to gender differences in access to inputs, resources, and services. This study also demonstrated gender differences in access to purchased inputs. For example, more male than female farmers were more likely to utilize pesticides, fertilizers and improved seed, all of which have a direct effect on yield. Inherent differences in male and female farmers' access to labor, which is also demonstrated in this study, limits the implementation of some agricultural practices, directly affecting productivity. Livestock units and farm size, on the other hand, represent a farmer's asset base and ability to afford new technologies. This implies the need to address gender differences in access to resources and inputs.

Farmers' most common sources of agricultural advice were: extension officer, TV/Radio and community exchange, and there were no reported gender differences in access to information sources. This is contrary to past research that shows that extension services are generally geared towards giving advice to male rather than female farmers.^38^ Other results^39^ are in line with this study as they report no significant differences in quality of advice given to male and female farmers at plant clinics in a study conducted in Ghana and Sri Lanka. This may be due to the availability of other non‐traditional approaches e.g. mass media and social networks, that may have reduced the gender divide in access to information. However, the extent to which male and female farmers implement agricultural advice and practices is limited primarily by the lack of access to actionable information.^40^ This implies the need to package information on fall armyworm management practices in gender‐appropriate formats, utilizing existing communication channels.

Results of this study are based on socio‐economic survey and farmer estimates of yield loss, which was also conditional on perceived crop damage/pest severity. While results are in tandem with other surveys undertaken in different countries in Africa estimating maize yield loss due to fall armyworm in the range of 22–67%,^10^, ^24^ other researchers using different methods (field scouting and harvesting of quadrants) estimate yield impact due to fall armyworm in Africa at about 9%.^41^ This estimate is much lower than what has been reported by socio‐economic surveys. To compound this, some recent studies, have demonstrated that in some genotypes, the damage by fall armyworm does not necessarily lead to serious injury to the crop to the extent that yield is highly impacted.^11^, ^42^ These authors suggest that maize plants are able to compensate for foliar damage incurred over a short period of time. Lima and Silva^43^ reported severe yield losses only when the whorl was destroyed. These assertions to some extent resonate with results of this study, where farmers reported nearly 50% of their cropped maize affected by the fall armyworm, yet estimated yield loss was less than one‐third. This potentially presents a limitation in existing yield loss estimation methods, and for this study as well, implying the need to enhance socio‐economic models to include other variables directly associated with yield loss e.g. genotype, cropping patterns, pest management regime, and presence of other biotic and abiotic stressors.

## 5 CONCLUSION

The results from this study are based on a combination of farmers' perceptions of fall armyworm infestation, their management practices, and estimated plot level maize yield loss. Farmers demonstrated an awareness of fall armyworm and could correctly identify it by its morphology or feeding habits. The larval form, identified by the farmer as a caterpillar, was the most prevalent pest stage observed. Pest management was mainly based on pesticide use, which was in part motivated by the free supply from the government in response to the outbreak. Increased fall armyworm prevalence has potentially intensified smallholder dependence on pesticides, which has implications for human health and environmental safety. This study, however, shows that farmers also employed a range of cultural and physical practices, based on indigenous knowledge e.g. hand picking of egg masses and caterpillars, and application of ash/sand on the larvae, some with considerable levels of success. Evidence from previous studies has also shown the effectiveness of some of these as well as other agronomic management practices, with relative affordability and less risk to health and environment compared to pesticide use. This offers opportunities for promotion (through farmer education) of alternative management practices, although validation by research is needed before wide‐scale promotion. Farmers attributed the current maize yield loss primarily to fall armyworm. The perceived, marginal decline in fall armyworm infestation and severity in the subsequent season, however, did not necessarily translate to better yields. This is attributed to the presence of other abiotic stressors, which underscores the need to tackle the fall armyworm invasion by providing integrated pest management solutions that also augment crop growth and response to other environmental stresses. Across gender and age categories, there were no observed significant differences in knowledge and awareness of fall armyworm, though management practices differed. This may be attributed to gender differences in access to resources and inputs, which were also responsible for the significant yield differences by male and female farmers. This suggests the need to integrate gender in agricultural programs and research paying attention to gender differences in access to resources, inputs, and labor. The majority of farmers had access to an external source of information, including extension officers, radio/TV or an informed neighbor or family member. Farmer information networks can further be exploited to share information on sustainable fall armyworm management practices. Packaging of information into farmer‐friendly formats disseminated through various channels will be key.
